# Emerging Roles for the Immune System in Traumatic Brain Injury

**DOI:** 10.3389/fimmu.2016.00556

**Published:** 2016-12-05

**Authors:** Celia A. McKee, John R. Lukens

**Affiliations:** ^1^Department of Neuroscience, Center for Brain Immunology and Glia, School of Medicine, University of Virginia, Charlottesville, VA, USA

**Keywords:** traumatic brain injury, neuroinflammation, cytokine, inflammasome, innate immunology, neuroprotection, microglia, neurodegeneration

## Abstract

Traumatic brain injury (TBI) affects an ever-growing population of all ages with long-term consequences on health and cognition. Many of the issues that TBI patients face are thought to be mediated by the immune system. Primary brain damage that occurs at the time of injury can be exacerbated and prolonged for months or even years by chronic inflammatory processes, which can ultimately lead to secondary cell death, neurodegeneration, and long-lasting neurological impairment. Researchers have turned to rodent models of TBI in order to understand how inflammatory cells and immunological signaling regulate the post-injury response and recovery mechanisms. In addition, the development of numerous methods to manipulate genes involved in inflammation has recently expanded the possibilities of investigating the immune response in TBI models. As results from these studies accumulate, scientists have started to link cells and signaling pathways to pro- and anti-inflammatory processes that may contribute beneficial or detrimental effects to the injured brain. Moreover, emerging data suggest that targeting aspects of the immune response may offer promising strategies to treat TBI. This review will cover insights gained from studies that approach TBI research from an immunological perspective and will summarize our current understanding of the involvement of specific immune cell types and cytokines in TBI pathogenesis.

## Introduction

Traumatic brain injury (TBI) affects millions of people worldwide every year, and current estimates from the World Health Organization (WHO) suggest that TBI will be the third leading cause of death and disability by the year 2020 (1). In the US alone, upwards of 1.7 million Americans seek medical treatment for some form of brain trauma each year (2, 3), and nearly 2% of the American population, or approximately 5–6 million people, currently suffer from TBI-related disabilities (4). TBI is a particularly serious threat to health in newborns, children, the elderly, military service personnel, and athletes involved in contact sports. Trauma to the brain can result in persistent and debilitating impairments in cognition, sensory function, mental health, and motor function. Furthermore, TBI-induced inflammation and pathology have been strongly linked to increased risks of developing numerous neurological disorders including anxiety, depression, PTSD, Alzheimer’s disease (AD), chronic traumatic encephalopathy (CTE), Parkinson’s disease, and amyotrophic lateral sclerosis (ALS) (5–10).

Despite being a prevalent and pressing global medical issue, there are currently no FDA-approved therapeutics to treat TBI. In recent years, mounting evidence from both TBI patients and animal models of brain injury implicate dysregulated immune responses in the potentiation of TBI-induced neurological dysfunction and brain pathology (11–16). For instance, elevated cytokine production is one of the strongest prognostic indicators of poor clinical outcomes in TBI (17–21), and brain trauma has been shown to induce immune-mediated inflammatory responses that can last for years post-injury (22, 23).

In addition to providing vital protective measures against pathogens and tumors, the immune system is also centrally involved in the restoration of tissue homeostasis following injury. Critical functions that are carried out by the immune system in response to injury include the sequestration of tissue-damaging irritants, engulfment and disposal of cellular debris, and the promotion of the wound-healing response. Tissue damage that results from trauma, ischemia-reperfusion injury, metabolic distress, and environmental irritants provokes the release of damage-associated molecular patterns (DAMPs) [e.g., ATP, reactive oxygen species (ROS), damaged mitochondria, and necrotic cells] and alarmins [e.g., interleukin (IL)-1α, IL-33, HMGB1]. The recognition of DAMPs and alarmins by immune receptors then stimulates the local production of cytokines and chemokines at the site of injury, which subsequently coordinates the activation, expansion, and recruitment of immune cells to areas of tissue damage.

**Figure 1:**
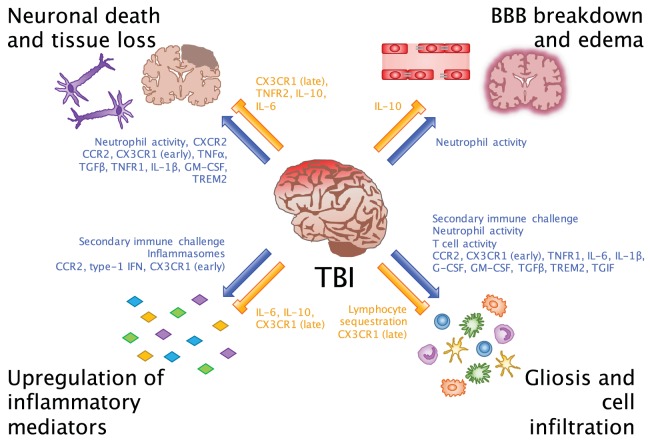
**Beneficial and detrimental roles for the immune system in TBI**. Common consequences of neuroinflammation after TBI include neuronal death and tissue loss, BBB breakdown and edema, upregulation of inflammatory mediators, and gliosis and cell infiltration. Researchers have evaluated these processes in order to understand which inflammatory cells and molecules potentiate (blue arrows) and inhibit (orange bars) the inflammatory environment of the brain. While we are beginning to link certain cells and molecules to their beneficial and detrimental effects in CNS injury, an important takeaway from these findings is that facilitators of inflammation may be involved in multiple processes at different points in time after injury.

Brain trauma results in two phases of tissue injury. The first round of injury is a direct result of exorbitant mechanical impact to the brain tissue. The aftermath of a severe blow to the head results in immediate neuronal and glial cell death, axonal injury, disruption of the blood–brain barrier (BBB), edema, and the release of DAMPs and excitotoxic agents (24). The immune response to TBI is intended to promote neuroprotection and repair, but can become maladaptive if dysregulation occurs. Whether the immune response contributes to repair or further destruction ultimately depends on the nature, duration, and magnitude of the immunological events that develop in response to brain injury. If not properly controlled, the immune system can provoke a secondary phase of tissue damage and neuroinflammation. In contrast to the acute nature of the primary brain injury, the secondary tissue damage generally results in a diffuse, long-lasting injury. The fundamental role that the immune system plays in driving the secondary phase of tissue damage following brain trauma has led many to believe that immunomodulatory approaches may offer a much-needed strategy to treat TBI. In this review, we discuss how aspects of the immune response can influence clinical outcomes following TBI. In particular, we highlight recent findings from experimental models of TBI that define central roles for individual immune cell types and cytokines in TBI pathogenesis (Figure 1; Table 1).

**Table 1:** **Key immune mediators involved in TBI pathogenesis**.

Cell types	Mediators	Functions
Neutrophils	CXCR2 (C–X–C motif chemokine receptor 2)	Chemokine that mediates neutrophil migration
	NE (neutrophil elastase)	Enzyme released by neutrophils to degrade extracellular matrix
Macrophages and microglia	CD11b (cluster of differentiation 11b)	Integrin that regulates migration of immune cells through tissues
	CCR2 (C–C motif chemokine receptor 2)	Chemokine receptor that coordinates monocyte chemotaxis
	CX3CR1 (C–X3–C motif chemokine receptor 1)	Chemokine receptor mediating macrophage and microglia migration
	IBA1 (ionized calcium-binding adapter molecule 1)	Calcium-binding protein associated with microglia and macrophage activation
T cells	Rag1 (recombination activating gene 1)	Enzyme that is required for B and T cell development
	IL-4 (interleukin 4)	Cytokine that aids in B and T cell proliferation and differentiation
Others	IL-1 (interleukin 1)	Pro-inflammatory cytokine that regulates transcription and production of multiple downstream inflammatory mediators
	Caspase-1	Enzyme that cleaves pro-IL-1β and pro-IL-18 to induce inflammation
	IL-18 (interleukin 18)	Pro-inflammatory cytokine that activates NK and T cells
	IL-6 (interleukin 6)	Pleiotropic cytokine that induces a multitude of inflammatory responses
	GFAP (glial fibrillary acidic protein)	Intermediate filament protein expressed by astrocytes
	TNFα (tumor necrosis factor α)	Pleotropic cytokine that can promote cell death, inflammatory cytokine production, and cell proliferation
	G-CSF (granulocyte colony-stimulating factor)	Stimulates proliferation and differentiation of hematopoietic cells as well as neural progenitors
	GM-CSF (granulocyte-macrophage colony-stimulating factor)	Promotes generation and activation of myeloid cells and neurons
	Type 1 IFN (type 1 interferon)	Regulates transcription of pro-inflammatory cytokines and chemokines
	IL-10 (interleukin 10)	Negatively regulates pro-inflammatory cytokine production
	TGF-β (transforming growth factor β)	Controls proliferation and differentiation of multiple immune cell types
	TREM2 (triggering receptor expressed on myeloid cells 2)	Activates myeloid cells upon sensing lipoproteins, may be involved in debris removal and cell survival

## The Kinetics of the Immune Response to Brain Injury

**Figure 2:**
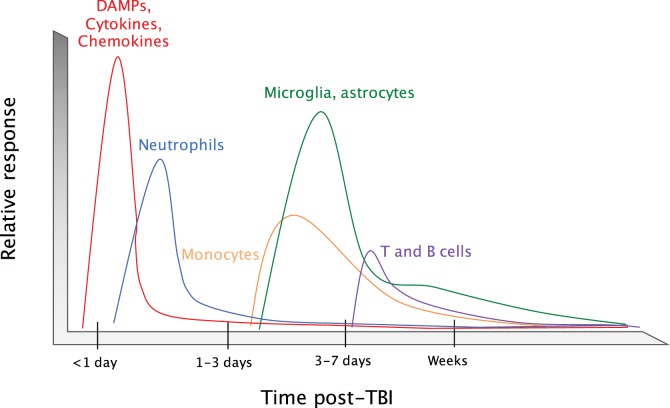
**Timeline of the immune response to TBI**. Upon an impact to the head, cellular damage results in the rapid release of damage-associated molecular patterns (DAMPs) that prompt resident cells to release cytokines and chemokines. These signals quickly call in neutrophils, which aid in the containment of the injury site and promote the removal of debris and damaged cells. As neutrophil numbers begin to decline after a period of days, infiltrating monocytes and activated glia begin to accumulate around the site of injury to perform reparative functions. Depending on the severity of the brain injury, T and B cells can also be recruited to sites of brain pathology at later time points in the response (3–7 days post-injury).

Upon brain injury, DAMPs and alarmins are released into the extracellular space where they can then signal through pattern-recognition receptors (PRRs) and cytokine receptors on CNS resident cells. This promotes the production of cytokines and chemokines that are involved in coordinating the recruitment of immune cells to sites of tissue damage (Figure 2). Neutrophils are the first immune cells that are recruited to the brain in response to trauma (25, 26). They first appear in the sub-arachnoid and vascular space surrounding the site of tissue damage within hours of injury. Neutrophils then begin to infiltrate into the brain parenchyma at 24 h post-injury (27). As the first responders, they play critical roles in the containment of the injury lesion and in the removal of cellular debris and damaged cells. Neutrophils predominate during the first days following injury; however, their numbers diminish greatly between days 3 and 5 post-injury. This time point coincides with the recruitment of other peripheral immune cells and the local activation of microglia and astrocytes. CCR2-expressing monocytes are the major immune cell population that infiltrates into the damaged tissue at days 3–5 post-injury, although T cells, natural killer (NK) cells, and dendritic cells (DCs) can also be detected around the injury site (15, 28–30). The coordinated production of chemokines following trauma orchestrates the recruitment of immune cells to areas of brain injury. The major chemokine pathways that are involved in mobilizing immune cells to brain damage have been comprehensively reviewed recently (11, 31–33) and will not be covered in great detail in this review. By 2 weeks post-injury, the brain is largely devoid of any infiltrating immune cells. However, activated microglia and astrocytes and elevated levels of inflammatory cytokines can be detected for months to years following brain injury (22, 23, 34, 35). This is unlike what is seen following tissue damage in other peripheral organs, where tissue resident macrophages and stromal cells typically return to a resting or immunologically quiescent state within weeks post-injury. The existence of activated glial cells and aberrant regulation of cytokine expression for months to years post-TBI suggests that the immune response to TBI can persist for long periods beyond the initial trauma.

## Involvement of Immune Cell Types in TBI

### Neutrophils

Considering their role in vascular permeability and edema in peripheral tissues, neutrophils have largely been implicated in BBB breakdown and edema in TBI. However, it is still unclear how their activity is related to these processes. Early TBI papers agree that neutrophils can accumulate at sites of injury within hours post-injury (26, 36) and that the number of neutrophils that are recruited to sites of brain trauma typically correlates with the severity of the injury (37). However, studies on the role of neutrophils in mediating BBB breakdown, edema, and neurodegeneration have been inconclusive. Although neutrophilia has been reported to coincide with BBB breakdown and neurodegeneration, these processes seem to be disconnected spatially and temporally from the invasion of neutrophils (26). Furthermore, efforts to deplete neutrophils have been unsuccessful in linking them to loss in BBB integrity (36), which was thought to be the event responsible for subsequent edema and neuronal death.

Due to these early findings, researchers began to think of neutrophil activity and tissue edema as having important consequences independent of BBB breakdown. Kenne et al. used an anti-Gr-1 antibody to deplete neutrophils in a cortical controlled impact (CCI) model and found that neutrophil depletion led to decreased edema for at least 48 h after injury, but did so without ameliorating BBB permeability (38). Neutrophil depletion was associated with decreased numbers of apoptotic cells, reduced macrophage/microglia activation in the cortex, and mitigated tissue loss. These data are similar to results from CXCR2 knockout mice, which were used by Semple et al. to reduce CXCR2-mediated infiltration of neutrophils after TBI (39). These mice did show reduced neutrophil infiltration into the brain, but BBB breakdown appeared similar to wild-type mice. While they also showed significantly less cell death within the lesion, this did not have an impact on functional outcome. Collectively, these two studies suggest that neutrophil depletion may have neuroprotective effects in TBI without necessarily being linked to BBB breakdown.

Further research into the importance of neutrophil activity post-TBI has begun to characterize the mechanisms involved in neutrophil-mediated neurodegeneration at early time points. For instance, Semple et al. used neutrophil elastase (NE) knockout mice in a CCI model to investigate how neutrophil effector functions contribute to secondary tissue damage and neurological dysfunction following brain trauma (40). They found that NE-deficient mice exhibit significantly diminished edema at 24 h after injury. However, this was not associated with reductions in neutrophil numbers or decreased production of matrix metalloproteinase-9 (MMP-9), which is known to regulate neutrophil migration by promoting extracellular matrix breakdown and/or through the modulation of chemokine activity. NE knockout mice also had reduced numbers of apoptotic neurons as well as lower heme-oxygenase levels in the hippocampus at 24 h after injury, indicating attenuated cell death and a less severe oxidative state. However, these early neuroprotective effects did not prevent cortical or hippocampal volume loss in the long-term, which may explain why NE deficiency was not found to improve behavioral performance at 2 months post-injury. These findings suggest that NE activity contributes to injury-induced edema and early neurodegeneration.

Thus, it is becoming clearer that neutrophils are linked to cerebral edema and neuronal death in TBI, but the relationship between neutrophil activity and BBB breakdown is not as clear as previously thought. It is likely that the distinct differences between the BBB and other vascular barriers outside the brain mean that the BBB structure has a different relationship with neutrophils that remains to be elucidated. In future studies, it will be important to investigate whether vascular edema in TBI is directly responsible for releasing cytotoxic substances that cause neuronal death following TBI or whether neutrophils and other inflammatory cells within the parenchyma are the primary source of neurotoxic factors that promote cytotoxic edema and early neurodegeneration in TBI.

### Macrophages and Microglia

There has been tremendous interest in defining the discrete roles of macrophages and microglia in TBI. Activated microglia and macrophages release pro- and anti-inflammatory factors that can signal to resident and peripheral cells to promote or resolve the inflammatory response to trauma. Chronically activated microglia and macrophages have been found in rodent models and humans after TBI (22, 23, 41, 42) and are considered to be one of the hallmarks of unresolved inflammation that may have long-term consequences (43, 44).

Groups have utilized different methods to deplete microglia and macrophages *in vivo* in order to characterize their roles in TBI-induced neuroinflammation, tissue damage, and neurological dysfunction. Two of these methods use targeted depletion of CD11b-expressing cells with transgenic CD11b-TK (thymidine kinase) and CD11b-DTR (diphtheria toxin receptor) mice (45, 46). While both methods were effective in reducing their target cell types post-TBI, neither attenuated signs of tissue damage such as axonal injury and lesion size. However, it should be noted that both of the treatment approaches that were used to deplete CD11b-expressing cells in these studies were found to cause inflammation even in uninjured mice. Therefore, it is likely that triggering inflammation before injury had an effect on the outcomes that were observed in these studies.

The chemokine receptor CCR2 plays critical roles in the recruitment of monocytes/macrophages to the brain, and, as a result, suppression of CCR2 signaling is often exploited to reduce the effects of infiltrating monocytes/macrophages in TBI studies. Numerous recent reports have shown that abrogating CCR2-mediated events can markedly limit both TBI-induced neuroinflammation and cognitive decline. For instance, Morganti et al. found that the CCR2 antagonist CCX872 reduces accumulation of peripheral macrophages in the brain and alters the regulation of several pro- and anti-inflammatory cytokines as well as NADPH oxidase (NOX2) production after CCI (47). These effects were associated with less severe hippocampal-dependent cognitive dysfunction. Similarly, CCR2 deficiency in another CCI study reduced numbers of infiltrating monocytes and rescued long-term spatial learning and memory deficits in the Morris water maze (MWM) test (48). Another group disrupted CCR2 activity by knocking in a red fluorescent protein (RFP) protein at the *Ccr2* gene locus in mice. In their studies, they found that impaired CCR2 signaling prevents monocyte recruitment into the brain and reduces cavity volume and axonal pathology following fluid percussion injury (FPI) (49). Taken together, these studies indicate that inhibition of CCR2-mediated cell infiltration limits neurodegeneration and neurological decline following brain trauma.

A recent study by Zanier et al. used CX3CR1 knockout mice to disrupt CX3CL1 chemokine signaling in order to understand its importance in controlling myeloid cell activity in TBI (50). After receiving a CCI injury, CX3CR1 knockout animals showed neurological protection 4 days following TBI. However, while wild-type mice returned to pre-injury levels of neuroscore performance by 5 weeks post-injury, CX3CR1-deficient mice still exhibited appreciable impairments in neuroscore performance at this time point. This decline in neuroscore performance at later time points in *Cx3cr1^−/−^* mice was associated with persistent neuronal death and an overall decrease in neuronal numbers. Further investigation into the effects of disrupted CX3CR1 signaling on macrophages and microglia showed that these cell types exhibit a more protective, anti-inflammatory phenotype in injured CX3CR1-null mice than seen in injured controls at early time points. However, at 5 weeks post-TBI, CX3CR1-deficient mice showed signs of elevated myeloid cell activation as compared to wild-type animals. Taken together, these results indicate that while early CX3CR1 signaling may have detrimental effects, this signaling is necessary at later time points post-brain injury to prevent long-term inflammation and cognitive impairment.

Another issue facing the TBI field is how to best define inflammatory cell types. Using principal component analysis (PCA) and microarray analysis of brain macrophages, Hsieh et al. found that the subset of macrophages expressing the M2 (alternatively activated macrophages)-associated marker arginase-1 (Arg1) had a distinctly different transcriptional profile from arginase-1-negative cells, but that the genes they expressed after TBI did not match traditional M2 markers (51). They found that while Arg1^+^ and Arg1^−^ macrophages expressed a variety of M1 and M2 markers, they differed distinctly in their chemokine profiles and several genes involved in injury protection and wound healing. These data indicate that delineating macrophages by an M1 (classically activated macrophages) or M2 phenotype in TBI obscures other macrophage subsets that may have distinct roles in the injury response.

Emerging data also suggest that macrophage phenotypes may be more flexible than once thought. Wang et al. set out to characterize the timeline of M1 and M2 macrophage/microglia activity after CCI. By tracking M1 macrophages/microglia with the marker CD16/32 and M2 macrophages/microglia with CD206, they found that at 3 and 7 days after injury the majority of Iba1^+^ cells assumed an M1 phenotype, yet at day 5 there was a rise in M2 macrophage/microglial cell numbers (52). This shift from an M1 state to an M2 phenotype and back may provide protection from possible detrimental effects of a prolonged state of either phenotype. The authors also found that white matter injury correlated with M1 cells, peaking at 3 and 7 days.

Clarifying the activation timeline and phenotypes of macrophages and microglia will likely be important in understanding how unresolved inflammation can lead to long-term detrimental consequences. An emerging body of literature is beginning to define how microglia and macrophages can be primed by TBI and generate exaggerated immune response and functional deficits upon secondary immune challenge. For example, injection of LPS at 30 days after injury in an FPI model induced more robust inflammatory cytokine production by CD11b-expressing cells in TBI animals than in controls (53). This was associated with decreased social exploratory behavior at 24 h after LPS injection as well as depressive behaviors. This same group found that secondary immune challenge also caused learning and memory deficits that could be linked to TBI-mediated microglia priming (54). These data indicate that at long-term time points, when behavioral deficits appear to have normalized following brain injury, a second immune challenge can produce further cognitive decline.

Taken together, these studies provide examples of how the TBI field is beginning to characterize macrophage and microglia migration, activation, and priming in relation to functional deficits after TBI. An important consideration for this field is that many authors choose to study both macrophages and microglia as a combined population, acknowledging that it is difficult to distinguish them within an inflammatory context using current markers, such as CD11b, CD45, CX3CR1, and IBA1. However, considering the importance of these cells in both the short and long-term inflammatory states, more specifically targeted techniques would help to define their discrete roles. In addition, considering the timeline of their activation, adapting methods to study macrophage and microglia signaling over the acute and chronic phases of TBI will be necessary in order to uncover time-dependent beneficial and detrimental effects as well as identifying effective therapeutic windows.

### T Cells

The kinetics of T cell infiltration have been described in TBI patients and animal models (55–57), but it still remains unclear what role(s) they play in brain trauma-associated wound-healing responses. In a study by Weckbach et al., *Rag1^−/−^* mice were used to investigate how the absence of B and T cells influences brain pathology and neurological impairment following weight drop-induced TBI (58). Surprisingly, lacking the adaptive arm of the immune system did not appreciably affect neurological outcome, BBB integrity, pro- or anti-apoptotic mediators, hippocampal architecture, or astroglial activation in these studies.

In a separate study, Mencl et al. used the sphingosine-1-phosphate receptor agonist and lymphocyte sequesterer FTY720 to inhibit T cell migration to the brain following TBI (59). While FTY720 did decrease the numbers of circulating lymphocytes, it did not provide any protection to TBI animals in terms of lesion volume, neuroscore, apoptotic neurons, BBB maintenance, or edema. However, FTY720 was found to reduce the numbers of neutrophils and macrophages/microglia in the ipsilateral hemisphere at 1 day after injury. Thus, future investigations should evaluate the ability of T cells to regulate the infiltration of other immune cells into sites of brain injury.

In future studies, it will be important to move away from methods that promote global defects in T cell responses and consider more specific effects of T cell subsets on TBI progression. In other models of CNS injury, T cells have been found to confer neuroprotection (60–62). For example, Walsh et al. recently reported that protection after spinal cord injury (SCI) is guided by specific T cell-derived cytokines, particularly IL-4 (63). Their interest in IL-4 stemmed from the observation that T cells within the site of injury were the major producers of IL-4 in their model and that functional recovery was markedly delayed following SCI in IL-4 knockout mice. They found that reconstituting *Rag1^−/−^* mice with IL-4-deficient T cells prior to injury did not lead to functional recovery, but transfer of wild-type T cells did. In addition, a MyD88-dependent Th2 skew of T cells was necessary to produce IL-4 and induce elevated neurotrophin signaling and axonal outgrowth both *in vitro* and *in vivo*. This insight into T cell subsets in injury recovery may apply to the TBI field, and thus warrants more specific investigations.

## Inflammatory Mediators in TBI

### Interleukin-1

Interleukin-1 is a potent pro-inflammatory cytokine that has been implicated in numerous inflammatory and neurological disorders. Secretion of IL-1 must be tightly regulated in the brain, as unchecked IL-1 production has been shown to provoke neuroinflammation and neurodegeneration. There are two distinct forms of IL-1 – IL-1α and IL-1β – both of which can induce similar levels of inflammatory signaling following engagement of IL-1 receptor (IL-1R). Although IL-1α and IL-1β evoke almost identical downstream inflammatory responses, their expression patterns and requirements for activation differ greatly. IL-1α is constitutively expressed by all nucleated cells, and secreted full-length IL-1α can transmit inflammatory signaling without the need for further modification or processing. In contrast, IL-1β is generated as a biologically inactive pro-form protein that requires cleavage to elicit its inflammatory properties and secretion (64). Caspase-1 activation in inflammasome complexes has emerged as a major mechanism for both IL-1β cleavage and IL-1α release (14, 65–69), although recent studies have also begun to identify additional inflammasome-independent pathways that promote IL-1 production (64, 70–72).

Interleukin-1β is one of the most frequently measured cytokines in the TBI literature, and it has been shown to be increased after TBI in humans and mouse models (46, 47, 53, 73–83). During neuroinflammation, IL-1β is known to have profound effects on BBB permeability, glial activation, immune cell recruitment, and neurodegeneration (84–86) and is likely one of the first immune mediators as it peaks early after injury. IL-1β is known to strongly enhance inflammatory responses following TBI, and this has led many to postulate that IL-1 production may negatively impact clinical outcomes following brain trauma (75).

Recently, progress has been made using methods to neutralize or antagonize the activity of IL-1β in TBI. In two different studies, Clausen et al. administered an anti-IL-1β neutralizing antibody to CCI-injured animals through 14 days after TBI (87, 88). In these studies, IL-1β neutralization led to a decrease in the numbers of microglia/macrophages, neutrophils, and T cells in the brain, especially at 7 days after injury. Although they did not report appreciable differences in motor coordination performance during the rotarod test, they did observe better performance during learning trials in the MWM, as well as decreased tissue loss at experimental endpoints for anti-IL-1β-treated animals.

In other models of CNS injury, IL-1α upregulation precedes that of IL-1β and IL-1α deletion limits neuronal damage and promotes accelerated functional recovery (89). IL-1α has also been suggested to jump start an inflammatory loop that is sustained and enhanced upon the upregulation of IL-1β, accounting for the excess of IL-1 signaling commonly seen in inflammatory states (90). Thus, future studies should investigate the effects of IL-1α separately from IL-1β, as their baseline expression, regulation, and secretion differ significantly and may thus control the kinetics of inflammation in different ways.

In humans, the recombinant IL-1 receptor antagonist anakinra is currently being tested to treat severe TBI, as it has shown some promise in the treatment of stroke (91). Helmy et al. used anakinra with PCA analysis to demonstrate that IL-1 signaling is a pivotal upstream regulator of TBI-induced cytokine production (92), which, they showed in a later trial, may lead to a shift in macrophages to express higher levels of pro-inflammatory cytokines such as granulocyte-macrophage colony-stimulating factor (GM-CSF) and IL-1β (93). This result is somewhat surprising considering the antagonistic effect on IL-1 signaling anakinra would be expected to have, but it suggests that further exploration into the mechanisms involved as well as delineation of the distinct functions of IL-1α and IL-1β during neuroinflammation will likely yield critical insights into the regulation of TBI pathogenesis by IL-1 signaling.

As described briefly above, inflammasome signaling has emerged as a major mechanism involved in IL-1 production. Inflammasomes are multiprotein complexes that coordinate caspase-1-mediated inflammatory cytokine production and cell death. Recent studies have shown that aberrant regulation of inflammasome signaling is a major driver of inflammation and pathology in multiple models of tissue damage, including stroke, macular degeneration, and renal ischemia (16, 66, 73, 94, 95). Inflammasomes consist of a sensor molecule such as a Nod-like receptor (NLR) or a pyrin/HIN domain-containing protein (PYHIN) family receptor, the adaptor protein ASC (apoptosis associated speck-like protein containing a CARD), and caspase-1. To date, five receptors – NLRP1, NLRP3, NLRC4, AIM2, and PYRIN – have been discovered to promote inflammasome signaling. Following the detection of their cognate danger- or pathogen-associated triggers, inflammasome-associated NLRs and PYHIN family receptors promote rapid inflammasome complex formation. The coordinated assembly of this multiprotein inflammasome platform promotes activation-inducing auto-cleavage of caspase-1. Activated caspase-1 can then cleave both pro-IL-1β and pro-IL-18, which is required to elicit their inflammatory properties and for their secretion. Bioactive caspase-1 also provokes pyroptosis, which is a gasdermin D-mediated inflammatory form of cell death that is associated with the release of the pro-inflammatory alarmins IL-1α and HMGB-1 (96, 97).

Since the finding that inflammasome proteins are upregulated after TBI in human patients (98), significant attention has been paid to identify the inflammasome-associated signaling events that are engaged in response to brain trauma (14, 99, 100). Inflammasome literature has identified the expression of NLRP1, NLRP2, and NLRP3 as well as AIM2 inflammasomes in microglia, neurons, and astrocytes in the CNS (100–104). Furthermore, recent studies in CNS injury models have uncovered critical roles for inflammasome signaling in driving inflammatory responses following tissue damage in the CNS. For instance, SCI leads to the upregulation and assembly of NLRP1 inflammasome components in spinal cord neurons (105). Moreover, neutralizing anti-ASC antibody treatment was also found to improve histopathological and functional outcome following SCI in these studies. In stroke models, methods to reduce inflammasome signaling, such as anti-NLRP1 neutralizing antibodies and caspase-1 inhibitors, as well as NLRP3, ASC, NLRC4, and AIM2 knockout mice, have all shown signs of improved functional recovery and reductions in inflammasome signaling (73, 103, 106, 107). Similarly, in an intracerebral hemorrhage model, both small interfering RNA and a selective inhibitor of the purinergic receptor P2X7R, which has been shown to promote NLRP3 activation in some experimental settings (108), limited inflammasome activation and led to neuroprotection (109). Considering the consistent benefits of inhibiting inflammasome components across these models, the inflammasome provides a tempting target for alleviating CNS injury.

Additional insights into the timing and importance of inflammasomes in CNS injury have been gained from recent TBI studies. In an FPI model, inflammasome components, such as ASC and caspase-1, were shown to be upregulated in cortical neurons for up to 24 h post-injury (110). Co-immunoprecipitation of inflammasome proteins also demonstrated that NLRP1 and ASC could be detected in multiprotein complexes in the brain. Treatment with an ASC-neutralizing antibody reduced caspase-1 activation and IL-1β production while also decreasing lesion volume, suggesting beneficial effects of targeting inflammasome activity. Liu et al. also recently showed that TBI results in upregulated expression of NLRP3, ASC, and caspase-1. Moreover, they report that the expression of these inflammasome-associated proteins can remain elevated out to 7 days post-injury (111). Importantly, inflammasome components in this model localized not only to neurons, but also to astrocytes and microglia, suggesting a wide range of inflammasome activation across cell types. Measurements of IL-1β and IL-18 protein levels also demonstrated that while IL-1β peaks around 6 h after injury and subsequently decreases over time, IL-18 expression remains elevated through 7 days after injury. In agreement with these findings, a separate study also reported elevated IL-18 production for at least a week post-TBI in both humans and experimental animals (112). These data suggest that early inflammasome production of IL-1β may be involved in acute inflammation and tissue damage, while inflammasome-driven IL-18 may contribute to the perpetuation of TBI-induced inflammation. It should be noted, however, that in a more recent study neither NLRP1 nor ASC knockout mice exhibited any improvements in lesion volume, histopathology, cell death, or motor function following CCI injury (81). It is possible that differences in the extent of caspase-1 abrogation and/or the timing of inflammasome inhibition or differences in injury models may help explain the disparate results that were reported in these studies.

Although key roles for inflammasomes have been clearly identified in other models of sterile inflammation and trauma, the specific contributions of inflammasome activation to TBI pathogenesis have only recently been investigated and multiple questions remain. For instance, although the formation of inflammasome complexes has been reported following TBI, the roles that specific inflammasomes play in driving TBI-associated pathology and neurological dysfunction have not been studied in great detail in animal models. In addition, the individual contributions of inflammasome-derived cytokines (i.e., IL-1α, IL-1β, and IL-18) and caspase-1-mediated cell death in TBI pathogenesis still remain poorly characterized. The major cell types in which inflammasomes operate to promote TBI progression have also not been formally defined to date. The genetic targeting of inflammasome signaling components in mice has aided in the discovery of critical roles for inflammasomes in other models of sterile inflammation. Future *in vivo* TBI studies that utilize these genetic tools should help to more fully characterize the contributions of specific aspects of inflammasome signaling in brain trauma.

### Interleukin-6

Interleukin-6 has frequently been associated with TBI outcome in humans, but it is unclear whether its role is primarily beneficial or detrimental. Microdialysis fluid detection of parenchymal IL-6 production has been associated with improved survival in TBI patients (21). However, more recent evidence points to a detrimental role for IL-6 in TBI (113). In these studies, plasma levels of IL-6 were shown to be significantly higher in severe TBI patients over moderate TBI patients. Both subacute and chronic serum levels of IL-6 have been associated with unfavorable short and long-term outcomes (75). In separating human patients by high or low cerebrospinal fluid (CSF) IL-6 trajectory, high trajectory patients are much more likely to have unfavorable clinical outcomes (76). Thus, while the role of IL-6 in TBI is still somewhat unclear, data from TBI patients indicate that IL-6 is consistently upregulated after TBI and can remain elevated in chronic stages, making it a potentially important mediator of long-term outcome.

Early animal studies verified that IL-6 is elevated in CSF and serum after TBI (114). Evidence from IL-6 knockout mice has also confirmed it as a pro-inflammatory cytokine that recruits activated glia and immune cells to sites of injury. Indeed, genetic ablation of IL-6 in cryolesioned mice resulted in fewer reactive astrocytes and macrophages and increased neuronal death (115). Conversely, overexpression of IL-6 in astrocytes enhanced recruitment of glia and immune cells to the lesion site and decreased both oxidative stress and neuronal death (115, 116). These studies suggest that IL-6’s role in inducing inflammation and glial scar formation is important in reducing prolonged cell death. A later CCI study also pointed to beneficial effects of IL-6 by showing that its deficiency leads to significantly poorer performance on behavioral tests as well as higher IL-1β protein levels in the cortex, suggesting that IL-6 may be an important regulator of IL-1β in TBI (117). However, a more recent study using a weight drop model showed that systemic neutralization of IL-6 mitigates some of the inflammatory and behavioral effects of hypoxia on exacerbating post-injury responses, implying that reducing the inflammatory response induced by IL-6 can indeed provide neuroprotection and lead to better outcome (118). When considering these types of studies, it is important to keep in mind the difference between complete or partial removal of a gene and/or its product. It is likely that some level of IL-6 is necessary to produce an inflammatory state that positively affects outcome such that either complete elimination or overexpression of IL-6 can be detrimental.

### Tumor Necrosis Factor α

Early work on the role of tumor necrosis factor (TNFα) in TBI mouse models suggested that it has early deleterious effects after TBI while exhibiting more protective effects in chronic stages (119). However, other work suggested that TNFα is necessary to protect from early mortality within a week of injury (120). Regardless of these contradictions, literature on TNFα in TBI consistently shows an upregulation of this cytokine after injury (74, 75, 113, 121), suggesting an important role for TNFα in both the acute and chronic phases.

The importance of TNFα early after injury was recently confirmed in a weight drop model. In this study, mice receiving a TNFα inhibitor at 1 and 12 h after injury showed improved cognitive performance 1 week post-injury, but mice administered the inhibitor at 18 h post-injury did not, implying a very short window for TNFα-targeting therapeutics after TBI (122). Further investigation of the mice given the inhibitor at 1 h showed fewer apoptotic neurons and less astrogliosis at 72 h after injury in both the cortex and dentate gyrus. This study outlines a 12-h window after injury during which the detrimental effects of TNFα may be attenuated, and points toward a tentative link between TNFα and prolonged astrogliosis and neuronal death.

Aside from defining the timing of TNFα activity in TBI, it will be important to elucidate the pro-apoptotic and pro-survival pathways in which it participates following brain trauma. In conjunction with its role as a major inflammatory switch, TNFα is known to induce both cell proliferation and apoptosis through several signaling pathways. While its activation of transcription factors, such as NF-κB and AP-1, can lead to transcription of inflammatory and apoptotic mediators, signaling through death receptors to activate caspases can also play an important part in determining cell death or survival. A recent article by Longhi et al. showed that separate deletion of either TNF receptor 1 (TNFR1) or 2 (TNFR2) can have opposite effects on cell survival and behavioral deficits (123). Using p55 (TNFR1) and p75 (TNFR2) knockout mice in a CCI model of TBI, they showed that TNFR1 deletion attenuated neuroscore deficits through 4 weeks post-injury and led to a shift to pro-survival signaling along with attenuated neuronal death and smaller lesion volume. TNFR2 knockout had the opposite effect in worsening neuroscore with no signs of pro-survival signaling or protection from cell and tissue loss. The TNFR1 knockout mice also showed a smaller area stained for CD11b as well as a higher area stained for Ym1, a marker for anti-inflammatory macrophage phenotypes, compared to the TNFR2 knockout group, suggesting opposite roles for the two receptors in recruiting inflammatory macrophages and microglia to the site of injury. These data agree with a paper by Yang et al. in which TNFR2/Fas knockout mice showed worse motor and cognitive performance after CCI TBI, although in that study neither TNFR1 or TNFR2 knockout alone had an effect on lesion volume or the number of dead cells in the cortex (124). Together, these studies indicate that the TNF receptors may play different roles post-injury, with TNFR2 providing a neuroprotective role and TNFR1 playing a detrimental one.

An important consideration about TNFα signaling is that due to the much wider expression of TNFR1 across cell types as well as its ability to respond to both forms of TNF (both soluble and transmembrane), this receptor could have more potent inflammatory consequences than its counterpart TNFR2. In addition, it has been shown that TNFR1 can signal through NF-κB, JNK, and caspase-mediated apoptotic pathways, while it is more common for TNFR2 to engage NF-κB and PI3K to induce pro-inflammatory and pro-survival signaling (125, 126). Thus, consolidating seemingly contradictory evidence for the role of TNFα in TBI with regards to cell death and clinical outcome will likely involve understanding the conditions under which its various forms and receptors participate in different survival or death pathways and the timeline on which this signaling can occur.

### Granulocyte Colony-Stimulating Factor/Granulocyte Macrophage Colony-Stimulating Factor

Both granulocyte colony-stimulating factor (G-CSF) and GM-CSF are involved in the expansion and mobilization of immune cells from the bone marrow and act as key cytokines in the inflammatory response. Interestingly, some recent evidence suggests that both G-CSF and GM-CSF may play a protective role in TBI. A recent paper using G-CSF administration after a CCI injury showed that G-CSF injection improves cognitive recovery and increases neurogenesis in the hippocampus (127). This was accompanied by higher activation of astrocytes and microglia as well as higher levels of brain-derived neurotrophic factor (BDNF) and glial cell line-derived neurotrophic factor (GDNF), indicating that G-CSF may regulate production of neurotrophic factors by activated glia post-TBI to promote neurogenesis. Similarly, a study using GM-CSF knockout mice showed that GM-CSF deficiency in TBI results in more cognitive deficits with higher tissue and neuronal loss after FPI (128). GM-CSF knockout mice also showed reductions in astrogliosis, which may suggest that GM-CSF plays a role in activating astrocytes to protect cells and boost tissue repair. Understanding how both of these molecules interact with glia to promote neuronal protection and regeneration may elucidate how other neuroprotective processes may involve glial functions.

### Type 1 Interferon

Increasing evidence points toward central roles of type 1 interferon (IFN) signaling in inflammatory CNS disorders and age-related cognitive decline (129–132). A recent study by Karve et al. is one of the first to investigate the involvement of type 1 IFN signaling in TBI pathogenesis (82). They found that deficiency in type 1 IFN signaling produced by either type 1 IFN receptor (IFNAR) knockout or an IFNAR blocking antibody reduces lesion volume. This neuroprotection was associated with a shift toward more anti-inflammatory cytokine signaling; however, this also coincided with increased GFAP and IBA1 staining. In addition, using a bone marrow chimera, they found that IFNAR deficiency in hematopoietic cells alone was sufficient to confer lesion volume protection and elevated GFAP and IBA1 staining. Importantly, they also showed that brain trauma in humans promotes enhanced expression of type-1 IFN, which suggests that type-1 IFN signaling may potentially influence clinical outcome in TBI patients.

### Interleukin-10

Interleukin-10 has been shown to be elevated in TBI patients (78, 133–135) and has been associated with unfavorable outcome and mortality (75, 134, 135). Despite these associations, Chen et al. found a role for IL-10 in conferring neuroprotection with hyperbaric oxygen (HBO) treatment (80). They found that the protective effects of HBO in TBI included reduced lesion volume and edema, improvements in cognitive performance, and the dampening of pro-inflammatory cytokine production in the cortex. It also led to a shift from apoptotic to cell survival pathways and greater BBB integrity. This wide range of positive effects was diminished in IL-10-knockout mice, and IL-10 injection by itself improved lesion volume, edema, and cognitive outcome in both wild-type and IL-10-knockout animals. This indicates an important protective role for IL-10 in TBI as well as a way to induce its production through HBO treatment. It is possible that the association between IL-10 (and likely many other cytokines) and poor outcome is primarily due to a widespread upregulation of cytokines after TBI, and that a more informative approach to understanding the role of IL-10 after brain injury involves dissecting its specific roles in damage responses.

### Transforming Growth Factor β

Transforming growth factor β (TGFβ) increases acutely in the serum and CSF of TBI patients (136). Several mediators of TGFβ signaling have been shown to be upregulated in TBI models (79, 137, 138). For example, transforming growth factor beta-activated kinase 1 (TAK1) was shown to increase in expression and is detected in cortical neurons and astrocytes after weight drop TBI (79). Inhibition of TAK1 signaling improved neuronal survival and motor function and also decreased NF-κB activity and inflammatory cytokine release. Transforming growth-interacting factor (TGIF), a transcriptional co-repressor of TGFβ that can inhibit transcriptional activation of TGFβ, was demonstrated to be upregulated in TBI animals and localized to both neurons and microglia (138). Using small hairpin RNA to knockdown TGIF levels in the brain, the authors found that lower TGIF levels led to a decrease in infarct volume and microglia number around the lesion, as well as a change in microglia morphology. Knockdown of TGIF also improved motor function through 2 weeks after injury. These data indicate that mediators of TGFβ signaling can have important inflammatory consequences.

## Future Perspectives

As highlighted in this review, increasing experimental evidence indicates that the immune system can profoundly influence clinical outcomes following TBI. Importantly, various recent studies have shown that targeting immune signaling with genetic and pharmacological approaches can lead to significant improvements in neurological function and tissue repair post-TBI. Both neuroprotective and detrimental roles have been assigned to the immune system in TBI. Whether the immune response contributes to beneficial tissue repair or further brain damage largely depends on the nature, kinetics, and magnitude of the inflammatory response. Although targeting the immune system has emerged as an exciting potential strategy to treat TBI, there are numerous outstanding questions that need to be addressed to better characterize the involvement of immune signaling in TBI etiology and to realize the full potential of immune-based therapeutics.

For one, there is still an overall lack of consensus on the overarching roles that discrete immune cell types and pathways play in TBI. Future efforts are needed to help reconcile the biological reasons that account for the disparate results that have been reported on immune mediators in TBI by different labs. Much of the inconsistency in the literature can be attributed to the utilization of different approaches to induce brain trauma and modulate immune signaling between labs (Table 2). Pinpointing what is mechanistically responsible for the conflicting findings in the TBI literature will help to uncover the important nuances of the immune response to brain trauma and will aid in the identification of optimal therapeutic regimens to treat discrete types of CNS injury.

**Table 2:** **Genetic models used to characterize the role of immune cell types and signaling pathways in TBI**.

Cell type	Animal line/model	Purpose	Major findings in TBI animals	Reference
Neutrophils	IgM RP-3	Neutrophil depletion	No significant decrease in BBB permeability	(36)
	Anti-Gr1 antibody	Neutrophil depletion	Decreased edema, apoptosis, and microglia/macrophage activation, no significant changes in BBB integrity	(38)
	CXCR2 knockout	Reduce neutrophil infiltration	Decreased cell death, no significant changes in BBB permeability or behavior	(39)
	Neutrophil elastase knockout	Reduce neutrophil effector functions	Decreased edema and apoptotic neurons, but no decrease in tissue volume loss or behavioral improvement	(40)
Macrophages and microglia	CD11b-TK	Deplete CD11b-expressing cells	Reductions in microglia numbers in the brain, no improvement in axonal injury, treatment toxic at high dosage	(45)
	CD11b-DTR	Deplete CD11b-expressing cells	No change in lesion size, treatment caused inflammatory response without injury	(46)
	CCX872 (CCR2 antagonist)	Reduce CCR2 signaling functions	Reduced macrophages in the brain, altered pro- and anti-inflammatory cytokine expression, less cognitive dysfunction	(47)
	CCR2 knockout	Limit CCR2-mediated recruitment of monocytes	Reduced numbers of infiltrating monocytes, improved learning and memory	(48)
	CCR2^RFP/RFP^	Disrupt recruitment of monocytes	Reduced monocyte recruitment, cavity volume, and axonal pathology	(49)
	CX3CR1 knockout	Abrogate CX3CR1 signaling functions in macrophages and microglia	Short-term neuroprotection and lower inflammatory response, long-term functional impairments and elevated myeloid cell activation	(50)
T cells	Rag1 knockout	Genetic ablation of B and T cells	No changes in neurological outcome, BBB integrity, pro- or anti-apoptotic mediators, hippocampal architecture, or astroglial activation	(58)
	FTY720	Sequester lymphocytes and reduce their migration to the brain	Decreased circulating lymphocytes, decreased neutrophils and macrophages/microglia in ipsilateral hemisphere	(59)

**Inflammatory mediator**	**Animal line/model**	**Purpose**	**Major findings in TBI animals**	**Reference**

IL-1	Anti-IL-1β antibody	Blockade of IL-1β signaling	Reductions in macrophages/microglia, neutrophils, and T cell numbers in the brain, improvement in learning tasks, and decreased tissue loss	(87, 88)
	IL-1R antagonist	Neutralize IL-1	Higher expression of proinflammatory cytokines in macrophages	(93)
ASC	Anti-ASC	Limit inflammasome assembly	Reduced caspase-1 activation and IL-1β production, decreased lesion volume	(110)
	ASC knockout	Abrogate inflammasome assembly	No improvements in lesion volume, histopathology, cell death, or motor function	(81)
NLRP1	NLRP1 knockout	Prevent NLRP1 inflammasome assembly	No improvements in lesion volume, histopathology, cell death, or motor function	(81)
IL-6	IL-6 knockout	Ablation of IL-6 signaling	Fewer reactive astrocytes and macrophages, increased neuronal death	(115)
	IL-6 knockout	Ablation of IL-6 signaling	Poor behavioral performance, higher IL-1β levels in the cortex	(117)
	GFAP-IL-6 overexpression	Increase IL-6 expression in astrocytes	Greater recruitment of glia and immune cells to the lesion, decreased oxidative stress and neuronal death	(116)
	Anti-IL-6 antibody	Neutralize IL-6	Reduced some inflammatory and behavioral effects of post-injury hypoxia	(118)
TNFα	TNFα inhibitor post-TBI	Inhibit TNFα signaling	Early administration improved cognitive performance, and decreased neuronal apoptosis and astrogliosis	(122)
	TNFR1 knockout	Disrupt TNFα signaling through TNFR1	Improved neurological function and neuronal survival/lesion volume, decreased numbers of CD11b^+^ cells in the brain	(123)
	TNFR2 knockout	Reduce TNFR2 signaling	Worsened neurological function and no protection from tissue loss	(123)
	TNFR2/Fas knockout	Abrogate TNFα signaling through TNFR2	Impaired motor and cognitive performance	(124)
G-CSF	G-CSF injection post-TBI	Enhance G-CSF signaling	Improved cognitive performance and increased hippocampal neurogenesis, higher glial activation and production of BDNF and GDNF	(127)
GM-CSF	GM-CSF knockout	Disrupt GM-CSF signaling	Worsened cognitive deficits as well as cell and tissue loss, reduced astrogliosis	(128)
Type 1 IFN	IFNAR knockout or IFNAR blocking antibody	Block type 1 IFN signaling	Reduced lesion volume, more anti-inflammatory cytokine signaling, increased glial activation, these effects were hematopoietic cell-dependent	(82)
IL-10	IL-10 knockout, IL-10 injection	Modulate IL-10 signaling	Diminished protective effects of hyperbaric oxygen treatment, including lesion volume, edema, cognitive improvement, and decreased cytokine production in IL-10 knockout mice, while IL-10 injection improved these outcomes	(80)
TGF-β	TAK1 inhibition	Disrupt signaling downstream of TGF-β	Improved neuronal survival and motor function, decreased NF-κB signaling and inflammatory cytokine production	(79)
	TGIF shRNA knockdown	Ablation of downstream TGF-β signaling	Decreased infarct volume and microglia numbers, improved motor function	(138)
APOE	APOEϵ4 overexpression	APOEϵ4 overexpression	Worsened brain pathology, BBB breakdown, and neurological impairments	(156, 157)
TREM2	TREM2 knockout	Abrogate TREM2 signaling	Altered macrophage distribution, hippocampal neuroprotection, and fewer cognitive deficits	(83)

One thing that is largely agreed upon in the literature is that no two brain injuries are alike and that seemingly similar types of trauma can result in diverse clinical outcomes. Unfortunately, the reasons for the heterogeneity in disease sequelae and recovery time associated with comparable forms of trauma remain poorly understood. Heterogeneity in recovery time and extent of neurological dysfunction in the TBI patient population can only be partially explained by differences in brain injury severity and location. This has led to greater appreciation for the roles of host genetics, environmental factors, lifestyle choices, and previous TBI history in overall clinical outcome. It is currently difficult to predict how interplay between these diverse non-injury factors affect TBI pathogenesis, and future studies are greatly needed to uncover their influence on TBI. Unfortunately, current TBI treatment approaches do not fully take into consideration many of the non-injury factors that are described above. The utilization of immune-based biomarkers in the future may offer a strategy to improve the stratification and treatment of TBI patient groups. Recent advancements from experimental TBI models indicate that the nature and kinetics of the immune responses can vary depending on brain injury location and severity. Furthermore, immune responses are also significantly affected by environmental and lifestyle factors (e.g., diet, antibiotics usage, prescription, or recreational drug use), microbiome composition, individual genetic factors, and previous TBI history. Therefore, it is feasible that immune cell frequencies and cytokine production in patients may serve as valuable biomarkers to predict clinical outcomes, stratify patient groups, and to maximize therapeutic approaches to treat TBI.

Mounting epidemiological evidence indicates that TBI is a major risk factor for developing numerous neurological disorders including AD, ALS, CTE, and posttraumatic stress disorder (PTSD) (5–10, 139, 140) and also possibly MS (141–143). Although it is widely appreciated that TBI predisposes individuals to other neurological disorders, how TBI mechanistically contributes to CNS disease later in life still remains poorly understood. Dysregulated immune responses have been identified to play key roles in the pathogenesis of the majority of neurological disorders and mental illnesses that have been linked to TBI. As a result, it is tempting to speculate that the aberrant inflammatory conditions that are generated in response to brain trauma may set in motion a series of events that can contribute to the development of other neurological disorders over time. Indeed, recent advances have been made in characterizing how TBI can contribute to AD pathogenesis. These studies have shown that brain trauma can spur the aberrant release and deposition of both amyloid beta (Aβ) and tau (144). The accumulation of neurotoxic forms of Aβ and tau are believed to be major drivers of AD pathogenesis (145), and studies of postmortem brains following brain trauma indicate that Aβ and tau deposition are also hallmarks of TBI (144, 146–152). The mechanism(s) by which Aβ and tau promote AD is still a matter of great debate; however, emerging data clearly point to roles for Aβ- and tau-induced neuroinflammation in this process (153). Therefore, it is conceivable that neuroinflammation and neuronal damage that is incited by the deposition of Aβ and tau following TBI may instigate a pathological cycle of continued Aβ and tau release and inflammation that initiates early AD progression. Furthermore, targeting the hyperinflammatory responses that are generated in response to Aβ and tau accumulation post-brain trauma could help to limit the risk of AD development in TBI patients (154, 155).

Interest in the link between TBI and AD has also extended to some of the major genetic susceptibility factors that are associated with AD. Genome-wide association studies (GWAS) have determined that carrying an allele of APOEϵ4 (apolipoprotein E ϵ4) or a mutation in TREM2 (triggering receptor expressed on myeloid cells 2) is associated with significantly higher rates of AD in humans (156). Interestingly, recent reports suggest that manipulation of either TREM2 or APOE biology can also affect the severity of brain pathology and neurological dysfunction following TBI (83, 157). For instance, it was shown that TBI results in more severe memory and functional impairments in individuals who carry the APOEϵ4 allele than in people who possess other APOE alleles (154, 158). Moreover, transgenic overexpression of APOEϵ4 in mice was also found to cause worsened brain pathology, BBB breakdown, and neurological impairments following brain injury (159, 160).

**Figure 3:**
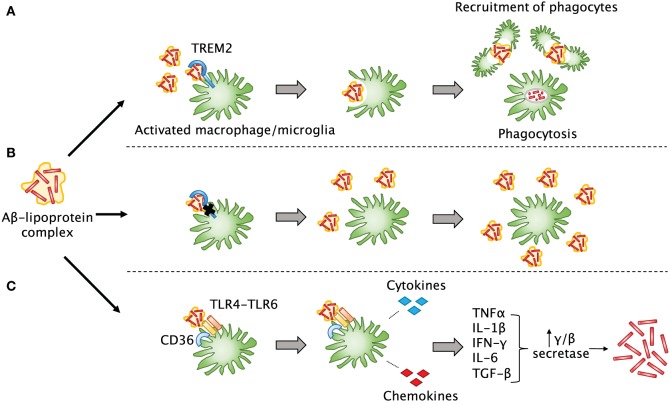
**The involvement of TREM2 in post-TBI amyloid beta clearance**. **(A)** Aβ released after TBI quickly forms into plaques and may bind to lipoproteins. TREM2 assists surrounding myeloid cells in sensing Aβ-lipoprotein complexes, engulfing and breaking down the Aβ, and recruiting other phagocytes to the site of injury. **(B)** In the case of TREM2 mutations or dysfunction, these cells may not be able to properly sense and clear Aβ or recruit other cells, so that more Aβ builds up over time and leads to plaque formation as seen in Alzheimer’s disease. **(C)** Another possibility is that in the absence of TREM2 signaling, other signaling pathways may predominate in myeloid cells. For example, when a complex of CD36 with a TLR4-TLR6 heterodimer senses Aβ, instead of phagocytosis, it leads to release of pro-inflammatory cytokines and chemokines that can induce upregulation of secretases in other cells, which are known to lead to increased production of Aβ.

TREM2 is an immunoglobulin-superfamily receptor that is predominantly expressed on myeloid cells including microglia, macrophages, and osteoblasts. Recent studies have shown that TREM2 is involved in the removal of debris, misfolded proteins, and phospholipids from the CNS (161). An important feature of both AD and TBI is that Aβ can accumulate into plaques (157), which are thought to lead to detrimental effects on neurological function if not cleared by phagocytes. TREM2 can help microglia and infiltrating macrophages detect lipoprotein-bound Aβ in the brain and trigger phagocytosis (162) and may also participate in maintaining the survival of these cells (163) (Figure 3). These activated cells can then recruit more phagocytes to the sites of Aβ accumulation. Downstream effects of this phagocytic response remain unclear, but multiple studies have agreed that TREM2 deficiency in various CNS disorders does lead to a decreased number of phagocytes, which likely impairs debris clearance (83, 164–166). In such cases of TREM2 mutation or dysfunction, it is possible that Aβ and other misfolded proteins may not only be insufficiently cleared, but may also accumulate faster. A recent article by Saber et al. used an FPI injury model to explore the effect of TREM2 on TBI-induced neuroinflammation, tissue loss, and neurological function (83). They found that TREM2-knockout mice did indeed show fewer macrophages throughout the brain, but more were present close to the site of injury. This was associated with hippocampal neuroprotection and fewer cognitive deficits in the TREM2 knockouts. Interestingly, sham TREM2-knockout mice appeared to have some differences from sham wild-type mice in certain behaviors. This paper shows that TREM2 is also likely to be important in TBI recovery; thus, it will be interesting going forward to elucidate the mechanisms by which it mediates debris clearance and neuroinflammation.

Despite the recent progress that has been made in characterizing how having a history of TBI can predispose individuals to AD, little is currently known about what biologically accounts for the increased risk of developing other neurological conditions in TBI patients. Immune dysfunction has been implicated in the pathogenesis of many of these CNS disorders that are more prevalent following brain trauma, including CTE, mental illness, and PTSD (167–169). Therefore, targeting the immune system following TBI may help to lower the risk of developing other neurological diseases later in life.

## Conclusion

Once considered a silent epidemic, TBI is now recognized as a serious threat to global human health. In recent years, considerable efforts and resources have been paid to reduce the rates and severity of TBI. Unfortunately, these preventative measures have been largely unsuccessful, and the number of individuals who sustain debilitating brain trauma each year continues to rise. As highlighted in this review, recent advancements in animal models of TBI clearly indicate that immune responses are centrally involved in the development of brain pathology and neurological dysfunction following TBI. Importantly, these emerging studies suggest that targeting the immune system could offer a much-needed therapeutic approach to treat TBI. Future studies that are geared toward further defining the major immunological pathways that influence TBI pathogenesis will lead to an improved understanding of brain injury etiology and will aid in the identification of novel immune-based TBI treatment strategies.

## Author Contributions

CM wrote the sections of the manuscript related to the involvement of immune cell types and inflammatory mediators in TBI; designed the manuscript; and created the figures. JL wrote the introduction, future perspectives, conclusion, and sections of the manuscript related to inflammasomes and the kinetics of the immune response to brain injury; designed the manuscript; and oversaw the entire process of manuscript preparation.

## Conflict of Interest Statement

The authors declare that the research was conducted in the absence of any commercial or financial relationships that could be construed as a potential conflict of interest.
